# Pet Macaques in Vietnam: An NGO’s Perspective

**DOI:** 10.3390/ani11010060

**Published:** 2020-12-30

**Authors:** Brooke Catherine Aldrich, David Neale

**Affiliations:** Animals Asia Foundation, Hong Kong, China; dneale@animalsasia.org

**Keywords:** exotic pets, primate trade, primate conservation, human-macaque interface

## Abstract

**Simple Summary:**

Macaques are regularly kept as pets in Vietnam, although the practice is illegal. This is substantially damaging to the individuals involved. In this article, we present the available data on the number of confiscations and releases of macaques throughout Vietnam over a five-year period (2015–2019). We examine this information alongside the data provided by Education for Nature Vietnam in a recent report on macaque possession cases. We also present insights from two key Animals Asia (a non-governmental organization) colleagues who work on the front lines of the macaque issue in Vietnam.

**Abstract:**

In this article, we attempt to characterize the widespread trade in pet macaques in Vietnam. Data on confiscations as well as surrenders, releases, and individuals housed at rescue centers across Vietnam for 2015–2019 were opportunistically recorded. Data comparisons between Education for Nature Vietnam and three government-run wildlife rescue centers show that at least 1254 cases of macaque keeping occurred during the study period, including a minimum of 32 Assamese macaques (*Macaca assamensis*), 158 long-tailed macaques (*Macaca fascicularis*), 291 Northern pig-tailed macaques (*Macaca leonina*), 65 rhesus macaques (*Macaca mulatta*), and 110 stump-tailed macaques (*Macaca arctoides*). A minimum of 423 individuals were confiscated, and at least 490 individual macaques were released. Three semi-structured interviews were conducted with two key Animals Asia (a non-governmental organization) colleagues and their insights are presented. Although we recognize that the data included are limited and can serve only as a baseline for the scale of the macaque pet trade in Vietnam, we believe that they support our concern that the problem is significant and must be addressed. We stress the need for organizations and authorities to work together to better understand the issue. The keeping of macaques as pets is the cause of serious welfare and conservation issues in Vietnam.

## 1. Introduction

The first step in solving a problem is to characterize it, which can be difficult when robust data are unavailable. In this article, we attempt to characterize the widespread trade in pet macaques in Vietnam. We stress the need for organizations and authorities to work together to better understand an issue that is currently the cause of serious welfare and conservation concerns in Vietnam.

There are five species of macaque native to Vietnam [1,2]. *Macaca assamensis* (Assamese macaques) are classified by the International Union for Conservation of Nature (IUCN) as Near Threatened (NT) and decreasing [3]. *Macaca fascicularis* (long-tailed macaques) have recently been reclassified from Least Concern (LC) to Vulnerable (VU) and are also considered to be decreasing [4]. An endemic subspecies of the long-tailed macaque, *M. f. condorensis*, lives only on the Con Dao islands off the south east coast of Vietnam [5]. *Macaca leonina* and *Macaca arctoides* (northern pig-tailed and stump-tailed macaques) are both classified as VU and decreasing [6,7]. Finally, *Macaca mulatta* (rhesus macaques) are classified as LC [8]. No current and comprehensive assessment of the conservation status of these species within Vietnam is available.

Possession of a macaque of any species is illegal in Vietnam unless a permit has been granted by the Forest Protection Department (FPD), whose remit includes enforcement of the legislation. Such permits are rarely granted and cannot be legally granted for the purposes of pet ownership.

Despite the legislation, macaque keeping is a relatively common practice. The purpose of this paper is to consolidate existing knowledge about the macaque pet trade in Vietnam and to highlight the need for improved understanding of relevant issues. This is essential to the development and implementation of feasible countrywide mitigation strategies.

It is a common assertion amongst those who work in relevant fields that in Asia, macaques are exploited as pets more frequently than most other nonhuman primate species (hereafter “primates”) [9]. At the same time, regardless of local legislation, macaques are protected less vigilantly than other primate taxa. Highly visible synanthropic macaques are often assumed to be abundant—even overabundant—but these assumptions can be wrong [10,11]. Possibly, for this reason, and despite a sizable body of academic and practical work on the Asian wildlife trade, little has been published specifically on the keeping of macaques in the region.

The unsuitability of primates as pets has been noted by primatological societies, veterinary bodies, conservationists, zoo and sanctuary professionals, animal welfare specialists, and medical professionals [12,13,14,15,16,17,18,19,20,21]. Such practitioners assert that although research pertaining specifically to the welfare of pet primates is scarce but see [22,23], the abundant psychological and physiological research that has been performed on laboratory primates is relevant to the welfare of those kept as pets [12,13,16,18]. Maternal deprivation and isolation from conspecifics, which are fundamental aspects of the primate pet trade, have been studied extensively in primates, largely macaques, since the 1960s. These studies demonstrate that maternal deprivation and social isolation can result in neophobia, persistent abnormal or stereotypical behaviors, anaclitic depression and withdrawal, and negatively affect plasma-cortisol levels, cell-mediated immunity, and survivorship [24,25,26,27,28,29,30]. Studies of captive primates in zoos and laboratories (where they are more likely to receive specialist care than in the hands of private individuals), demonstrate that abnormal behaviors and captivity-related complications are difficult to avoid, thus in less professional settings such problems are likely magnified [31,32,33,34].

In a study of ex-laboratory chimpanzees at a North American sanctuary [35,36], rescued individuals showed clear signs of what may be comparatively described as post-traumatic stress disorder (PTSD) in humans, according to the criteria set out in the Diagnostic and Statistical Manual of Mental Disorders (DSM-IV). A similar study found that ex-pet capuchin monkeys resident at a sanctuary in the UK showed symptoms of disorders homologous to PTSD, major depression, and generalized anxiety disorder, at significantly higher rates than zoo capuchins [37].

## 2. Materials and Methods

The Animals Asia Foundation works in three primary areas in Vietnam: ending bear bile farming, cat, and dog welfare, and captive wild animal welfare. On an ongoing basis, from 2017 onwards, D. Neale and Animals Asia staff in Vietnam have been opportunistically recording first-hand information and information from news articles and social media posts about confiscations and surrenders (hereafter “rescues”), transfers and releases of macaques in the country, and individuals housed at rescue facilities countrywide. Because this database is a live document and constantly updated, a copy was made on 23 September 2020. All Animals Asia macaque database information below is based on the contents of the database at that time. Some instances in the database were recorded retrospectively, reaching back as far as 2010. However, older instances were sporadically recorded and thus were disregarded. Data for 2020 are still being compiled. Accordingly, all data presented in Table 1 and Table 2 are based on the years 2015–2019.

Separately, complete macaque rescue numbers were obtained by Animals Asia staff directly from Phong Na Rescue Centre (PNRC) for 2015–2019, Hanoi Wildlife Rescue Centre (HWRC) for 2019, and Cat Tien Rescue Centre (CTRC) for 2018. These are government-run rescue centers. Animals Asia employs several animal welfare specialists to consult on husbandry at Vietnam’s rescue centers and so are involved in the recording of, and have access to, such data in cases where records are kept.

To obtain further insight into the Vietnamese trade in macaques as pets, three semi-structured interviews were conducted with two key members of Animals Asia’s Vietnam staff. Such qualitative methodology is increasingly common in primatological studies and can provide important nuance that may be difficult to otherwise capture [38]. Subjects gave their verbal informed consent before the interviews, which were conducted in accordance with the Declaration of Helsinki. Interviewee One is employed by Animals Asia to receive, track, and follow up on animal welfare complaints across Vietnam and is regularly involved in confiscations and transfers to rescue centers. Interviewee Two is a wildlife rehabilitator employed by Animals Asia to advise on welfare improvements at government-run rescue centers throughout the country and works directly with macaques in these centers. Interviews were conducted via Skype between July and September 2020.

For the purposes of this paper, the term “pet” is used broadly in concert with Serpell to imply “a blanket description for animals that are kept for no obvious practical or economic purpose” [39]. Defined thus, pet keeping comprises a wide array of different practices and motivations. Serpell also highlighted the contrast between “pets” and “companion animals” who are more valued, often kept inside the home, and treated as members of the family.

## 3. Results

**Figure 1:**
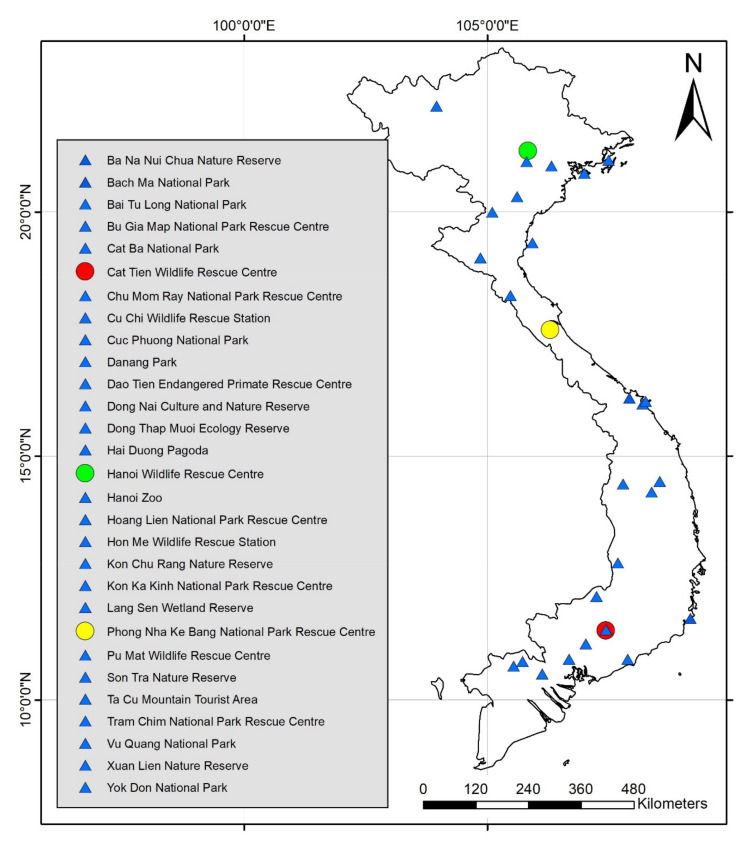
Locations of rescue center or release sites, Animals Asia macaque database. Colored circles represent rescue centers for which rescue data appear in Table 1. Blue triangles may represent rescue centers, release sites, or both.

The Animals Asia macaque database identified 29 sites that are either rescue facilities that house (or have housed) macaques or have served as release sites (or both) (Figure 1). It is assumed that the facilities listed are licensed by the relevant FPD to participate in such activities, but it must be noted that possession of such a license is not indicative of quality of care or good practice.

The numbers of rescues recorded in the Animals Asia macaque database and the numbers obtained from the three rescue centers (PNRC, HWRC, CTRC) are provided in Table 1, and are presented alongside the macaque possession cases made public by Education for Nature Vietnam (ENV) in July 2020 [40].

The total number of macaque cases recorded per species for 2015–2019 is shown in Table 2 for AAF, ENV, and PNRC. HWRC and CTRC are excluded from this table because data were unavailable for the full five-year period.

Information collected during the semi-structured interviews were unsuitable for quantitative analysis. Both interviewees believe that the macaque pet trade is of particular concern; that the number of individuals in need of rescue greatly exceed the number of actual rescues; that macaques are most often kept as novelties or attractions; and that education and resources are needed country-wide to address the problem. Their views are discussed in more detail in the next section.

## 4. Discussion

Records of macaque confiscations and releases in Vietnam are not kept centrally, and where they do exist, can be difficult to obtain. Although we report all available information, the data provided above are unlikely to represent the true extent of the issue. ENV, who have recently made public the possession cases listed in Table 1, define these cases as those logged in their Wildlife Crime Incident Tracking System, involving physical possession of one or more macaques. ENV states that there are likely hundreds of unreported cases [40]. Therefore, Table 1 represents an absolute baseline for the numbers of macaques kept as pets in Vietnam. ENV’s data are likely to be the more robust due to the nature of the work of their Wildlife Crime Unit, which exists “to facilitate and motivate public involvement in combating the wildlife trade while improving the effectiveness of law enforcement’s response to wildlife crime” [41]. If the data we compiled here are proportioned similarly to the actual number of kept macaques in Vietnam, then *Macaca leonina* (Northern pig-tailed macaques) are kept far more frequently (23.20–37.12% of cases) than Vietnam’s other macaque species. The least frequently kept species is *Macaca assamensis* (Assamese macaques) at between 0.88% and 7.57% of recorded cases (Table 2).

Many known cases involve unidentified macaque species (Table 2). ENV explain that this can arise from poor identification skills on the part of the public (where cases were reported but not followed up) and, less often, on the part of the local authorities involved in the case [40].

Animals Asia regularly receives reports from concerned members of the public about mistreated or illegally kept wildlife across Vietnam. According to Interviewee One, macaques are the most frequently reported animal, with reports occurring approximately every three to four weeks. Although such reports sometimes involve macaques kept by travelling circuses or substandard zoos, most often they involve macaques kept as pets. Pet macaques are normally kept in cages in gardens or in front of homes, hotels, or restaurants. Both interviewees believed that macaques are usually kept as a novelty or attraction, and that they are rarely kept inside the house.

Pet-keeping has been explored in an array of contexts, from classical antiquity, to tribal Amazonia, to contemporary Europe [42,43,44,45,46]. While motivations for keeping wild animals as pets have been studied, no specific research is available for the Vietnamese macaque trade. Studies in Mexico, Madagascar, Indonesia, and Russia reported such diverse motivations as: company [14,47]; entertainment [47,48]; to warn of intruders [47]; and companionship or comfort [14,48]. These studies also identified status [49] and attention [50] as common motives.

Reports received by Animals Asia about, such animals are investigated and passed on to the relevant authorities for follow-up. Interviewee One has observed, over the last five years, an increase in responsiveness to such reports. Interviewee One attributes this increase to public awareness campaigns run by non-governmental organizations (NGOs). Moreover, according to Interviewee One, in the past, action on the part of the authorities following a report was rare, and the macaques involved either remained in the hands of those keeping them illegally or became untraceable. In such cases, Interviewee One believes that it is likely that the people involved were warned that they should not have the monkey and so they sold, passed on, or released it. Currently, at least in some parts of the country, the relevant authorities are showing greater willingness to follow up on reports and to confiscate illegally kept macaques. However, ENV has stated that such enforcement is becoming a burden for the authorities [40].

Interviewee Two estimates that, on an average day, a rescue center might receive about three macaques, although at times, the numbers are far higher. Interviewee Two describes a “conveyor belt system”, recalling that one center released 22 macaques one day, only to receive 23 the next day. In another example, Interviewee Two and colleagues set out to collect one macaque but returned with three animals from three separate sites. Interviewee Two believes that the problem is worsening. Rescue centers across the country are filled beyond capacity. Because so many rescue centers have reached capacity, macaques are regularly released inappropriately or even immediately following confiscation. Such releases may be conducted without regard for the individuals’ ability to survive, or for the geological, ecological, or political suitability of the site. No disease screening is performed; no consideration is given to the potential for the release of habituated and confiscated macaques to exacerbate existing conflicts with humans in the area. Macaques are susceptible to various viral infections, theoretically including severe acute respiratory syndrome coronavirus 2 (SARS-CoV-2), the virus responsible for the COVID-19 pandemic [51,52]. In 2001, antibodies to measles, influenza, and parainfluenza viruses were found in macaques in Sulawesi, all of which can be debilitating or fatal to these primates [53], and which may have been introduced to wild macaque populations via the pet trade [48].

Apart from warehousing (a term coined to describe the lifetime housing of surplus zoo individuals in off-exhibit, often sub-par conditions) rescued individuals in overcrowded centers [54], releasing them, or leaving macaques in the control of those keeping them illegally, there are few solutions available to the authorities. While it could be argued that euthanasia is a welfare-friendly solution in the face of overcrowded rescue centers and large numbers of confiscated macaques, it is not a viable option: the cultural barriers are too high. Interviewees One and Two stress that reports of illegally kept macaques are usually made by local people, and if it were known that a macaque would be killed on confiscation, local people would likely be unwilling to report.

Conservation concerns may also present a barrier to the use of euthanasia. Many who study macaques have observed that while the synanthropic species are largely considered overabundant, this may be a product of their high visibility around areas where they have learned to exploit sources of food within human settlements. For example, the conservation status of the ubiquitous long-tailed macaque (*Macaca fascicularis*) has recently been raised from Least Concern to Vulnerable [10,55] suggesting that those species often perceived as overabundant may be, in fact, in decline [56].

Interviewees One and Two agree that despite improved enforcement, penalties (according to Interviewee One these range from a simple warning to a small fine) for those keeping pet macaques are too light to be deterrent. However, both believe that the most important aspects of solving the macaque problem lie elsewhere. Interviewee Two stresses the need for an increase in capacity at rescue centers, and the implementation of proper release protocols. Interviewee One believes that awareness and education programs, such as those run by Animals Asia and ENV, hold great potential for reducing the burgeoning trade in macaques as pets, and should be rolled out more widely.

## 5. Conclusions

The data compiled here cannot reasonably estimate the scale of macaque keeping in Vietnam. However, alongside the reports of those who are working in the field, it is apparent that there are large-scale negative consequences arising from the keeping of macaques as pets, including negative impacts on human and primate welfare and threats to local ecosystems. To resolve these problems, we propose that the Vietnamese authorities, non-governmental organizations, biologists, and conservationists should work together to accomplish the following:

1. Robust data on the present distribution and abundance of each of Vietnam’s five endemic macaque species are required. This will help to determine the urgency of the trade in pet macaques as a conservation issue.
2. Comprehensive records on confiscations and releases should be maintained by each confiscating authority. These records should be centrally located and broadly accessible. Reliable data on confiscations and releases are vital to recommendations below.
3. Intensive studies should be conducted on the scale of and reasons for macaque pet keeping. This information will inform and enable the development of the educational programs recommended below.
4. Improvements and training. Enforcement of the existing legislation requires confiscation of pet macaques. To safeguard animal welfare and enable confiscations to continue, rescue facilities must be expanded and improved. Animal husbandry and species identification training should be provided for rescue center workers and confiscating authorities.
5. Release protocols should be developed and implemented. If conducted appropriately, the release of rescued macaques could continue to make available valuable space in rescue facilities rather than causing further welfare and conservation issues.
6. Education and awareness campaigns should be rolled out countrywide. Non-governmental organizations, such as Animals Asia and ENV are already conducting such campaigns, with locally positive results. Such programs should be, supported, expanded, and rolled out countrywide as a long-term, preventative measure. Widespread education and awareness about the inappropriateness and illegality of keeping macaques as pets is likely the key to abating or ending the trade at its source.

## Figures and Tables

**Table 1:** Macaque rescues, possession cases, and releases 2015–2019.

Animals Asia Macaque Database = AAF Education for Nature Vietnam = ENV Phong Nha Rescue Center = PNRC Hanoi Wildlife Rescue Center = HWRCCat Tien Rescue Center = CTRC			AAF—Rescues	ENV— Possession Cases [29]	PNRC— Rescued	HWRC— Rescued	CTRC— Rescued	AAF— Released
2015	Assamese macaque	*Macaca* *assamensis*	11	2	1	-	-	2
	Long-tailed macaque	*Macaca* *fascicularis*	0	30	0	-	-	0
	Pig-tailed macaque	*Macaca* *leonina*	9	58	2	-	-	9
	Rhesus macaque	*Macaca* *mulatta*	10	15	5	-	-	4
	Stump-tailed macaque	*Macaca* *arctoides*	22	22	5	-	-	14
	Unknown macaque	*Macaca* spp.	0	131	0	-	-	0
2016	Assamese macaque	*Macaca* *assamensis*	10	4	1	-	-	8
	Long-tailed macaque	*Macaca* *fascicularis*	13	30	0	-	-	33
	Pig-tailed macaque	*Macaca* *leonina*	26	69	5	-	-	26
	Rhesus macaque	*Macaca* *mulatta*	8	5	4	-	-	9
	Stump-tailed macaque	*Macaca* *arctoides*	14	25	4	-	-	16
	Unknown macaque	*Macaca* spp.	0	95	0	-	-	0
2017	Assamese macaque	*Macaca* *assamensis*	4	3	4	-	-	6
	Long-tailed macaque	*Macaca* *fascicularis*	3	31	0	-	-	0
	Pig-tailed macaque	*Macaca* *leonina*	72	43	8	-	-	72
	Rhesus macaque	*Macaca* *mulatta*	14	7	10	-	-	10
	Stump-tailed macaque	*Macaca* *arctoides*	12	24	7	-	-	11
	Unknown macaque	*Macaca* spp.	0	122	0	-	-	0
2018	Assamese macaque	*Macaca* *assamensis*	4	1	1	-	0	3
	Long-tailed macaque	*Macaca* *fascicularis*	56	27	0	-	1	99
	Pig-tailed macaque	*Macaca* *leonina*	21	63	10	-	4	21
	Rhesus macaque	*Macaca* *mulatta*	18	6	10	-	0	18
	Stump-tailed macaque	*Macaca* *arctoides*	22	28	6	-	1	22
	Unknown macaque	*Macaca* spp.	0	100	0	-	0	0
2019	Assamese macaque	*Macaca* *assamensis*	3	1	2	13	-	18
	Long-tailed macaque	*Macaca* *fascicularis*	4	40	0	29	-	2
	Pig-tailed macaque	*Macaca* *leonina*	29	58	7	25	-	29
	Rhesus macaque	*Macaca* *Mulatta*	15	8	11	18	-	24
	Stump-tailed macaque	*Macaca* *arctoides*	18	11	5	10	-	30
	Unknown macaque	*Macaca* spp.	5	195	0	0	-	4
		**Total** **recorded:**	**423**	**1254**	**108**	95 (2019 only)	6 (2018 only)	**490**

**Table 2:** Macaque rescues (AAF, PNRC) and possession cases (ENV) by species.

Macaque Cases by Species 2015–2019	AAF	ENV	PNRC
*Macaca assamensis*	32	11	9
*Macaca fascicularis*	76	158	0
*Macaca leonina*	157	291	32
*Macaca mulatta*	65	41	40
*Macaca arctoides*	88	110	27
*Macaca* spp.	5	643	0

## Data Availability

The data presented in this study are available on request from the corresponding author. The data are not publicly available due to institutional restrictions.
